# Surgical outcomes between two endoscopic approaches for maxillary cysts

**DOI:** 10.1016/j.bjorl.2022.05.006

**Published:** 2022-06-02

**Authors:** Zhengcai Lou

**Affiliations:** Yiwu Central Hospital, Department of Surgery, Yiwu City, Zhejiang Province, China

**Keywords:** Maxillary sinus, Retention cysts, Middle Meatus Antrostomy, Inferior Meatal Antrostomy, Electrocoagulation

## Abstract

**Objective:**

To compare recurrence rates and symptomatic relief in symptomatic maxillary sinus Retention Cysts (RCs) between Middle Meatus Antrostomy (MMA) alone and Inferior Meatus Antrostomy (IMA) with basal mucosa electrocoagulation.

**Methods:**

Patients with symptomatic unilateral maxillary RCs were randomly allocated to MMA (n = 54) and IMA combined with mucosa electrocoagulation (n = 53) groups. Symptomatic relief, cyst recurrence, and closure of the antrostomy opening were compared at 12-months postoperatively.

**Results:**

Symptomatic failure occurred in 13 (12.1%) patients, including 9 (16.7%) MMA and 4 (7.5%) IMA patients; this difference was not statistically significant (*p* = 0.251). Postoperative cyst recurrence occurred in 16 (29.7%) and 1 (1.9%) patient in the MMA and IMA groups, respectively (*p* < 0.0001). Closure of the opening occurred in 7 (13.0%) and 17 (32.1%) patients in the MMA and IMA groups, respectively (*p* = 0.032). However, there were no significant pairwise correlations between closure of the opening and symptomatic failure or cyst recurrence.

**Conclusion:**

IMA combined with basal mucosa electrocoagulation and MMA alone provided similar symptomatic relief for symptomatic maxillary RCs, but IMA had shorter operation times and lower postoperative recurrence rates of RCs.

**Level of evidence:**

Level 1b.

## Highlights

• The headaches were the most common symptom of Maxillary Sinus (MS) Retention Cysts (RCs).
• 70.1% of RCs within MS were in the inferior wall.
• IMA combined with basal mucosa electrocoagulation and MMA alone provided similar symptomatic relief.
• IMA had shorter operation times and lower postoperative recurrence rates of RCs.
• There were no significant pairwise correlations between closure of the opening and symptomatic failure or cyst recurrence.

## Introduction

Maxillary Sinus (MS) Retention Cysts (RCs) are usually asymptomatic and often present as chance findings on imaging.^1^, ^2^ However, recent studies have demonstrated relationships between MSRCs and various symptoms, such as headaches, facial pain, nasal discharge, and postnasal drip.^3^, ^4^, ^5^, ^6^, ^7^ MSRCs are generally self-limiting, with spontaneous regression and disappearance rates of 17.6%–38%, but a few continue to gradually increase in size.^3^ Although some studies have demonstrated no significant correlations between cyst size and symptoms, surgical removal remains recommended for symptomatic RCs.^5^, ^6^, ^7^, ^8^, ^9^

Traditionally, symptomatic RCs have been treated by puncture and aspiration through the inferior meatus or ostium, or by removal using the Caldwell-Luc approach^8^, ^9^; however, these procedures have been replaced by endoscopic Middle Meatus Antrostomy (MMA) and Ostiomeatal Complex (OMC) removal.^4^, ^5^, ^7^ In recent years, some authors have reported that MMA does not provide adequate access to the entire MS and limited the use of some instruments, resulting in residual and recurrent lesions (e.g., inverting papillomas and fungal maxillary sinusitis),^10^, ^11^, ^12^, ^13^, ^14^ but Inferior Meatus Antrostomy (IMA) provides visibility and access to areas inaccessible with the MMA approach and reduces recurrence.^13^, ^14^ The objective of this study was to compare the recurrence rates and symptomatic relief of RCs between MMA alone and IMA combined with basal mucosa electrocoagulation.

## Methods

### Research ethics approval

This study was reviewed and approved by the Medical Research Ethical Committee of Yi Wu Central Hospital (nº 20111211). Informed consent was obtained from all participants.

### Patient selection

The present study was conducted in the Department of Otolaryngology, Head and Neck Surgery, Yi Wu Central Hospital between January 2012 and December 2019.Consecutive patients who presented with symptomatic unilateral MSRCs and were followed up for at least 12-months were included in the study. Patients were excluded if they met the following criteria: presence of bilateral MSRCs, concurrent chronic rhinosinusitis, substantial anatomical variations of OMC, and/or allergic rhinitis; requirement for regular decongestant use 1-month prior to surgery; and/or simultaneous septal or inferior turbinate surgery. Careful history assessment and physical examination, including rigid endoscopy, were performed for all participants. Patients were asked to grade their unilateral symptoms using a Visual Analogue Scale (VAS), with a score of 0 for absence of symptoms and a score of 10 for the maximum severity. For the purposes of this study, only values corresponding to the side with RCs were recorded.

**Figure 1:**
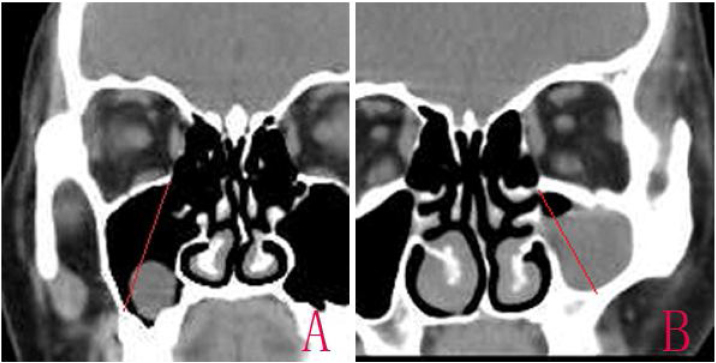
CT coronal scanning. The maxillary sinus diameter from the highest point of the superior wall to the lowest point of posterior lateral wall (A and B).

The diagnosis was confirmed based on the Computed Tomography (CT).^15^ The size of the cyst was measured in its greatest dimension, the antral size was measured in its diameter from the highest point of the superior wall to the lowest point of posterior lateral wall in CT coronal scanning, and the ratio of the RC size to antral size was calculated (Fig. 1). The operation time was recorded from nasal local anesthesia induction to RC removal.

### Randomization

Surgical type allocation was performed by the principal investigator, with the aid of a registered operating room nurse, using simple random sampling. Consecutive patients who met the inclusion criteria and consented were assigned random numbers that had been generated by SPSS Statistics software (ver. 19.0; IBM, Corp., Armonk, NY, USA); they were then allocated to either the MMA group or the IMA combined with basal mucosa electrocoagulation group. All procedures were performed endoscopically.

### Surgical technique

#### MMA

The natural MS ostium was identified and enlarged after uncinate process resection. The MMA size was as large as possible to facilitate access to the required maxillary regions. After satisfactory endoscopic visualization of the MS, the lesion was resected. The underlying cyst wall was removed using angled endoscopes, angled instruments, and curved microdebriders. Healthy MS mucosa was preserved.

#### IMA combined with basal mucosa electrocoagulation

Endoscopic IMA has been previously described by various authors.^10^, ^16^ An incision was made into the lateral wall of the inferior meatus using needle tip electrocautery; the incision was extended anteriorly in the sagittal plane up to the level of the anterior nasal spine. The lateral nasal mucosa was elevated using a suction elevator. The top and bottom of the mucosal flap were cut; the flap was prepared and inserted deep into the inferior meatus. The flap and the inferior turbinate were medialized together using a Freer elevator. The inferior opening of the nasolacrimal duct (Hasner’s valve) was identified, and the medial wall was penetrated approximately 5 mm posterior to this opening, using a 3-mm curved suction cannula. The small opening was widened using either cutting forceps posteriorly and a pediatric backbiter anteriorly, or drills (Fig. 1). The size of the opening was tailored according to the site and size of the cysts, with careful technique to avoid injuring the nasolacrimal duct.

**Figure 2:**
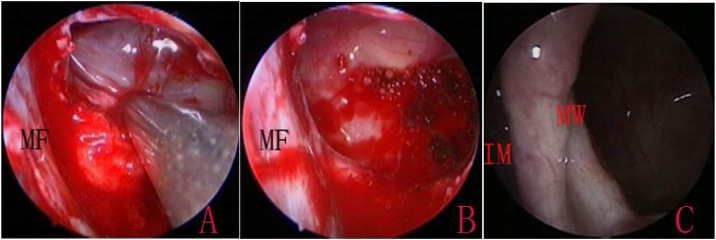
The retention cyst in the left lateral wall. Extraction of the cyst (A), cauterization of the base (B), and IMA opening at 12-months postoperatively (C). IM, Inferior Meatus; MF, Muco-periostal Flap; MW, Medial Wall. Black triangle indicated the retention cyst.

The MS walls were viewed using 0-degree and angled endoscopes. Following the confirmation of cysts, the cyst wall was exfoliated, and the cyst was extracted through the inferior meatus. For large cysts that could not be removed through the opening, an incision was made into the medial wall of the cyst to drain its contents prior to removal. The attachment sites of the cyst were then cauterized using an electrocoagulator, while the healthy mucosa was preserved (Fig. 2). The electrocoagulator used in this study was a disposable monopolar device, which consisted of a hand lever with a suction hole and an electrotome. The electrocoagulator was 25 cm in length and 2 mm in outer diameter, with a 14-cm hand lever; it could be curved to various angles, as required. After resection, the mucosal flap was replaced to cover the medial wall defect and the floor of IMA antrostomy, and the inferior turbinate was lateralized to its original position. No nasal packing was required.

#### Follow-up

Follow-up visits were scheduled at 3, 6 and 12 months after surgery; the opening was endoscopically examined at each visit. CT was performed if the opening had closed within 12 months postoperatively. Patients were asked to score their unilateral symptoms again on the VAS. For the purposes of this study, the VAS score obtained during the last follow-up visit was considered. If this score was ≥3, the case was considered a failure.

### Statistical analysis

Statistical analysis was performed using SPSS Statistics software (ver. 10.0). Results were expressed as means ± standard deviations and ranges. The VAS scores before and after ESS were compared using the Wilcoxon signed rank test. The preoperative CT scores, the RC diameters, the ratios of RC sizes to MS sizes, and the pre- and postoperative VAS scores were compared between the two groups using the Mann–Whitney *U* test. Fisher’s exact test was used to analyze the relationship between success rate and surgery type. Correlation analyses were performed using Spearman’s rank correlation coefficient. A *p*-value < 0.05 was considered statistically significant.

## Results

During the 7-year study period, 107 cases (41 men and 66 women) of unilateral symptomatic MSRCs were analyzed. Patients had a mean age of 43.8 years (range: 21–68 years). There were 39 (36.4%) left-sided and 68 (63.6%) right-sided RCs. Of the 107 patients, 46 (43.0%) were referred from the neurology department, 19 (17.8%) were referred from the stomatology department, and 42 (39.3%) presented directly to the otolaryngology department. Regarding the chief complaint, 71 (66.4%) patients presented with ipsilateral facial pressure or pain, or headache; 25 (23.4%) patients presented with nasal discharge and postnasal drip; and 11 (10.3%) patients presented with nasal obstruction. The mean duration of symptoms was 16.19 months (standard deviation: 7.53; range: 4–73 months).

### Endoscopic evaluation

A history of recurrent episodes of acute sinusitis, successfully managed with decongestants and antibiotics, was reported by 31 (29.0%) patients. The RC protruded into the middle meatus through the ostium and mimicked antrochoanal polyps in one patient. Another patient had a lesion that bulged toward the medial wall. There were no lesions bulging toward the nasal cavity or any other abnormal findings in the remaining patients.

The mean RC size was 23.13 mm (standard deviation: 6.24; range:15.58–51.38 mm), and the mean ratio of RC to antral size was 47% (standard deviation: 7.4; range: 38%–100%). The specific locations of RCs within the MS were as follows: inferior wall (n = 75; 70.1%), medial wall (n = 11; 10.3%), lateral wall (n = 8; 7.5%), anterior wall (n = 8, 7.5%), posterior wall (n = 3; 2.8%), and superior wall (n = 2; 1.9%).

The MMA and IMA groups comprised 54 and 53 cases, respectively. The baseline characteristics (demographic data, symptoms, RC sizes, ratios of RC to antral sizes, and percentages of cases with asthma and allergy) of the two treatment groups are presented in Table 1. The groups were well-matched in all baseline characteristics. The mean operation times were 38.61 ± 2.54 min and 26.74 ± 1.52 min in the MMA and IMA groups, respectively (*p* < 0.001).

**Table 1:** Baseline characteristics of the two treatment groups.

Parameter	MMA (n = 54)	IMA (n = 53)	*p*-Value
Age, years,	42.2 ± 1.6	44.9 ± 2.3	0.46
Sex (males/females)	10:44	11:42	0.96
Nasal side (Left:Right)	17:37	22:31	0.38
The duration of symptom, months, mean	17.16	15.94	0.12
Headache score, mean ± SD^a^	5.14 ± 1.28	5.19 ± 1.46	0.83
Facial pain/pressure score, mean ± SD^a^	5.38 ± 1.36	5.21 ± 1.17	0.75
Nasal discharge score, mean ± SD^a^	3.48 ± 1.71	3.81 ± 1.42	0.69
Nasal obstruction score, mean ± SD^a^	2.94 ± 1.35	2.91 ± 1.47	0.74
RC size, mm, mean ± SD^a^	22.8 ± 2.16	23.7 ± 1.89	0.63
Ratio RC size/antral size, mean ± SD^b^	46.93 ± 5.24	47.25 ± 3.17	0.91

SD, standard deviation; RC, retention cyst.

a Unilateral score on the RC side.

b Cyst size ×100/antral size.

### Postoperative outcomes

The mean follow-up duration was 13.9 months (range: 12–26 months). Patients were asked to rate their symptoms on the VAS at the follow-up visits. There was considerable symptomatic improvement after surgery in 87.9% (94/107) of the patients, with statistically significant decreases in unilateral VAS scores for headaches, facial pain and pressure, nasal discharge and postnasal drip, and nasal obstruction (Table 2).

**Table 2:** Symptomatic relief following surgery.

Symptom	Pre-	Post-	*p*-Value
Headache score, mean ± SD^a^	5.17 ± 1.26	2.24 ± 1.31	<0.0001
Facial pain/pressure score, mean ± SD^a^	5.29 ± 1.08	1.49 ± 1.26	<0.0001
Nasal discharge score, mean ± SD	3.64 ± 1.27	0.63 ± 0.93	<0.0001
Nasal obstruction score, mean ± SD	2.89 ± 1.46	0.91 ± 1.01	<0.0001

SD, standard deviation.

a Unilateral score on the retention cyst side.

There were 17 (15.9%) postoperative cyst recurrences, of which 16 (29.7%) were in the MMA group and one (1.9%) was in the IMA group (*p* < 0.0001). The recurrent RCs had the same location as the primary cyst in all 17 patients. There was symptomatic failure in 13 (12.1%) patients, including 9 (16.7%) in the MMA group and 4 (7.5%) in the IMA group; this difference was not statistically significant (*p* = 0.251). Among the 9 MMA patients with symptomatic failures, cyst recurrence occurred in 5 patients. Among the 4 IMA patients with symptomatic failures, there were no cyst recurrences. Symptomatic failure involved facial pain or pressure (n = 11; 84.6%), or postnasal drip (n = 2; 15.4%). None of the failed cases presented with nasal obstruction or discharge. The relationships between treatment type and symptomatic relief are presented in Table 3. We did not observe better outcomes with IMA, compared with MMA. The relationships of symptomatic relief with surgery type, RC size, and RC to antral size ratio are presented in Table 4. We found that symptomatic relief was not correlated with surgery type or cyst diameter.

**Table 3:** The association between treatment and symptomatic relief.

Symptom	MMA	IMA	*p*-Value
Headache score, mean ± SD^a^	2.17 ± 1.21	2.21 ± 1.64	0.84
Facial pain/pressure score, mean ± SD^a^	1.41 ± 1.17	1.38 ± 1.26	0.79
Nasal discharge score, mean ± SD	0.61 ± 0.84	0.59 ± 0.91	0.63
Nasal obstruction score, mean ± SD	0.88 ± 1.25	0.93 ± 1.12	0.81

SD, standard deviation.

a Unilateral score on the retention cyst side.

**Table 4:** Predictors of symptomatic success in maxillary cyst surgery.

Parameter	Success	Failure	
Surgery	45, MMA	9, MMA	0.25
	49, IMA	4, IMA	
RC size, mm, mean ± SD^a^	23.93 ± 2.41	22.85 ± 5.17	0.74
Ratio RC size/antral size, mean ± SD^b^	47.13 ± 2.85	45.93 ± 3.13	0.82

SD, standard deviation.

a Unilateral score on the retention cyst side.

b Cyst size ×100/antral size.

Endoscopy showed closure of the opening in 7 (13.0%) and 17 (32.1%) patients in the MMA and IMA groups, respectively (*p* = 0.032). Among the 7 MMA patients with closure of the opening, CT revealed cyst recurrence in one patient, while 3 patients had symptomatic recurrence (one case of postnasal drip and two cases of facial pressure/pain). Among the 16 MMA patients with cyst recurrence, closure of the opening occurred in one patient. Cyst recurrence occurred in the inferior wall in 12 (75.0%) patients, in the anterior wall in 3 (18.8%) patients, and in the medial wall in 1 (6.3%) patient. Among these 16 patients, 11 (68.8%) reported symptomatic relief, while 5 (31.1%) had symptomatic recurrence; the cysts were removed again using IMA. There were no significant pairwise correlations between closure of the MMA opening and symptomatic or cyst recurrence.

Among the 17 IMA patients with closure of the opening, no cyst recurrences were observed on CT, and only one patient had symptomatic recurrence of postnasal drip. The one case of cyst recurrence in the IMA group was located in the medial wall, close to the natural MS ostium; it was not correlated closure of the opening or symptomatic recurrence. The cyst was removed through the original IMA opening. There were no significant pairwise correlations between closure of the IMA opening and symptomatic or cyst recurrence. In addition, 76.5% (13/17) of the closures of the opening were in the medial MS mucosa and 23.5% (4/17) in the lateral nasal mucosa.

## Discussion

RCs are generally considered self-limiting, and most cases are treated conservatively. However, some RCs may cause clinical symptoms. Wang et al.^3^ reported nasal obstruction, nasal discharge, and headaches in 52.5%, 35.7%, and 2.5% of the MSRC patients, respectively. In a study of symptomatic RCs by Hadar et al.,^4^ headaches were reported by 63% of the patients. Albu et al.^5^ reported facial pressure or pain as the major symptom (60%) of RCs. In the present study, headaches were the most common symptom, and most patients were referred from the neurology department. We found that 43.0% of the patients initially presented to the neurology department for headaches, while 17.8% presented to the stomatology clinic with facial pain or pressure; these patients were subsequently diagnosed with RCs on CT.

Surgical removal is recommended for symptomatic RCs.^3^, ^4^, ^5^, ^6^, ^16^ In this study, all RCs were symptomatic and required surgical removal. This study demonstrated significant symptomatic improvement after surgical RC removal. Although establishing a relationship between symptoms and MSRCs is difficult,^17^ our results suggest that RC removal could provide symptomatic relief, in agreement with the findings in previous studies. Hadar et al.^4^ reported symptomatic improvement in 92% of symptomatic MSRCs after ESS. Albu^5^ reported symptomatic improvement in 88.8% of cases after MMA. In addition, Behrbohm and Sydow^18^ demonstrated that RCs > 1.5 cm in size significantly delay mucus transport within the MS; these RCs increase the risk of chronic maxillary rhinosinusitis. Cyst removal can restore the altered mucociliary clearance.^18^ Thus, surgical removal should be considered for symptomatic RCs.

This study found no significant correlations between symptomatic improvement and surgical type. Unsurprisingly, reduced intracystic pressures after surgical intervention led to symptomatic improvement. Similar to the results of previous studies, we did not find any pairwise relationships between symptomatic failure and RC size or the ratio of RC to antral size.^3^, ^5^ Further investigations are needed to determine whether the symptoms of RCs are related to anatomical variations of OMC. Bhattacharyya^15^ found no correlation between OMC obstruction and the occurrence of RCs. Albu^5^ also found no significant differences between the rates of OMC obstruction in control and cyst sides in symptomatic cases. In the present study, patients with substantial anatomical variations of OMC were excluded; no significant differences in OMC anatomies were found between groups. The OMC was not addressed in the IMA group. These findings suggest that RCs may be the main cause of the symptoms.

In recent years, some scholars have found that specific MS lesions (e.g., mucoceles, inverting papillomas, and fungal infections) have higher recurrence rates when treated using the MMA approach. IMA and modified IMA approaches have some advantages for these diseases, particularly for lesions on the inferior, medial, and lateral MS walls.^12^, ^13^, ^14^, ^18^ The MMA approach does not completely expose the MS walls and limits the use of certain instruments, such as radiofrequency ablation and drills; this increases the risk of recurrence.^13^, ^14^ Despite the enhanced field of view with extended MMA, it remains difficult to use rigid instruments at the anterior and inferior regions, as well as the prelacrimal and alveolar recesses. Beswick et al.^19^ demonstrated that despite the use of a combination of shavers with different angles, only 81% of the MS surface area could be accessed through a large MMA. Govindaraju et al.^20^ found significantly less residual disease in the anterior, anterolateral, and inferomedial aspects of the MS when IMA was performed, compared with MMA. An endoscope IMA approach provides unobstructed visualization of the inferior, medial, and lateral walls; it also facilitates instrumentation.

Previous studies have suggested that RCs are mostly located in the inferior half of the MS. Wang et al.^3^ reported that 70% of RCs were in the floor, 20.9% were in the lateral walls, and only 2% were in the superior wall. Albu^5^ reported that 70% of RCs were in the floor of the MS, while 25% were in the medial, lateral, or anterior wall. In the present study, 70.1% of RCs were in the inferior wall, 10.3% in the medical wall, while only 1.9% were in the superior wall. These RC locations make the IMA approach preferable. In addition, the medial wall of the MS is thin, particularly near the inferior turbinate, and provides easy access for the instruments. Usually, large IMA openings are not required to identify RC attachment sites. IMA provides a generally straight access route, which may shorten the operation time. This study found significantly shorter operating times when IMA was performed, compared with MMA.

The disposable monopolar electrocoagulation device can be curved as necessary to form different angles; it allows access to various areas of the MS. In this study, mucosal stripping of the attachment site was performed to remove the entire mucocele. The attachment areas were then cauterized using an electrocoagulator to achieve complete ablation of the cyst wall and preservation of the healthy mucosa, thereby avoiding recurrences. However, similar to the results of previous studies,^5^ we found that surgical outcomes were independent of surgery type. Notably, the MMA approach had higher recurrence rates, compared with the IMA approach, which could increase patient anxiety and medical costs.

The realization of the mucoperiosteal flap before antrostomy was made in the IMA technique. The mucoperiosteal flap should be carefully preserved and restored to cover the floor and the medial wall defect of IMA antrostomy, which is an important detail to prevent the postoperative occlusion of IMA. In present study, the persistent patency of IMA was obtained in 67.9% of IMA surgeries. Although the IMA openings had higher closure rates, compared with MMA openings in this study, the closure of the opening was not correlated with recurrences of cysts or symptoms. In the IMA group, four patients with symptomatic failure did not have cyst recurrence or antrostomy closure, while one patient with cyst recurrence did not have closure or symptomatic recurrence. Unfortunately, we noticed that most closures were caused by the medial MS mucosa, rather than the lateral nasal mucosa. This suggests that the medial mucosa of the MS opening also should be preserved and used to cover the lateral nasal wall, thereby preventing closure.

Theoretically, the presence of an open IMA is the potential mucus recirculation between inferior antrostomy and main Ostia of the MS, it could result in the persistence of symptoms, especially for the posterior drip. In this study, 4/53 IMA patients presented symptomatic failures, but only one patient had persistence posterior drip. Surprisingly, the patient showed the closure of IMA. Previous study suggested that the MS secretions will be transported toward the natural ostium even in the presence of a previously placed large and patent IMA.^21^ The IMA does not disturb mucociliary clearance and the post-operative nasal condition, on the contrary, it might contribute to improved mucociliary function.^12^, ^22^ Some scholars found that the double pathway yields good postoperative airflow, which enables a good outcome in a patient with fungus ball maxillary sinusitis.^23^ The limitations of this study were its small sample size and the short follow-up duration of 12-months.

## Conclusions

IMA combined with basal mucosa electrocoagulation and MMA alone provided similar symptomatic relief, but IMA had shorter operation times and lower postoperative recurrence rates.

## Conflicts of interest

The author declares no conflicts of interest.
